# Surgery for clinoidal meningiomas with cavernous sinus extension: Near-total excision and chiasmopexy

**DOI:** 10.1007/s00701-022-05281-z

**Published:** 2022-06-27

**Authors:** Daniele Starnoni, Constantin Tuleasca, Marc Levivier, Roy T. Daniel

**Affiliations:** 1 Department of Clinical Neurosciences, Centre Hospitalier Universitaire Vaudois (CHUV), Neurosurgery Service and Gamma Knife Center, Lausanne, Switzerland; 2 Faculty of Biology and Medicine (FBM), University of Lausanne (UNIL), Lausanne, Switzerland; 3 Signal Processing Laboratory (LTS 5), Ecole Polytechnique Fédérale de Lausanne (EPFL) , Lausanne, Switzerland

**Keywords:** Meningioma, Clinoid, Chiasmopexy, Radiosurgery

## Abstract

**Background:**

The main factors limiting the extent of resection for clinoidal meningiomas are cavernous sinus extension and vessel adventitia involvement. The proximity to the optic apparatus and the risk of radiation-induced optic neuropathy often prevents many surgeons from proposing adjuvant radiosurgery.

**Method:**

We describe a simple technical solution that is to place a fat graft between the optic apparatus and the residual tumor to maintain the distance gained at surgery and facilitates the identification of anatomic structures.

**Conclusion:**

This technique allows to deliver optimal therapeutic doses to the residue reduces the dose received by the optic nerve below 8 Gy.

**Supplementary Information:**

The online version contains supplementary material available at 10.1007/s00701-022-05281-z.

## Relevant surgical anatomy

The dura that covers the upper surface of the anterior clinoid process (ACP) extends medially to line the optic strut and form the antero-medial part of the distal dural ring (DDR) [6]. The dura of the DDR and the proximal dural ring (PDR) joins posteromedially to form a single dural layer that blends into the diaphragm sellae. The dural membrane, lining the lower margin of the ACP, extends medially to surround the ICA and forms the PDR and the carotid oculomotor membrane which forms the anterior part of the roof of the cavernous sinus (CS). The carotid collar, formed by the dura of the lower ring turning upward to surround clinoidal ICA, disappears posterior to the tip of the ACP; at this level the dura lining, the upper and lower surfaces of the ACP fuse into a single dural layer that forms the posterior part of the roof of the CS [6]. The dura that covers the anterior root of the lesser wing forms a dural fold, the falciform ligament that extends above the optic nerve (ON) just proximal to the nerve entry into the optic canal. The falciform ligament blends medially into the dura covering the planum sphenoidale. The intracranial portion of the nerve is directed posteriorly, superiorly, and medially toward the optic chiasm. The dural sheath around the ON blends smoothly into the periorbita at the anterior end of the optic canal. The ophthalmic artery is the first branch of the supraclinoid segment of ICA; it arises just distal to the DDR on the superior surface of ICA and courses forward and laterally to reach the optic canal [6].

## Description of the technique

### Surgical technique

**Fig. 1:**
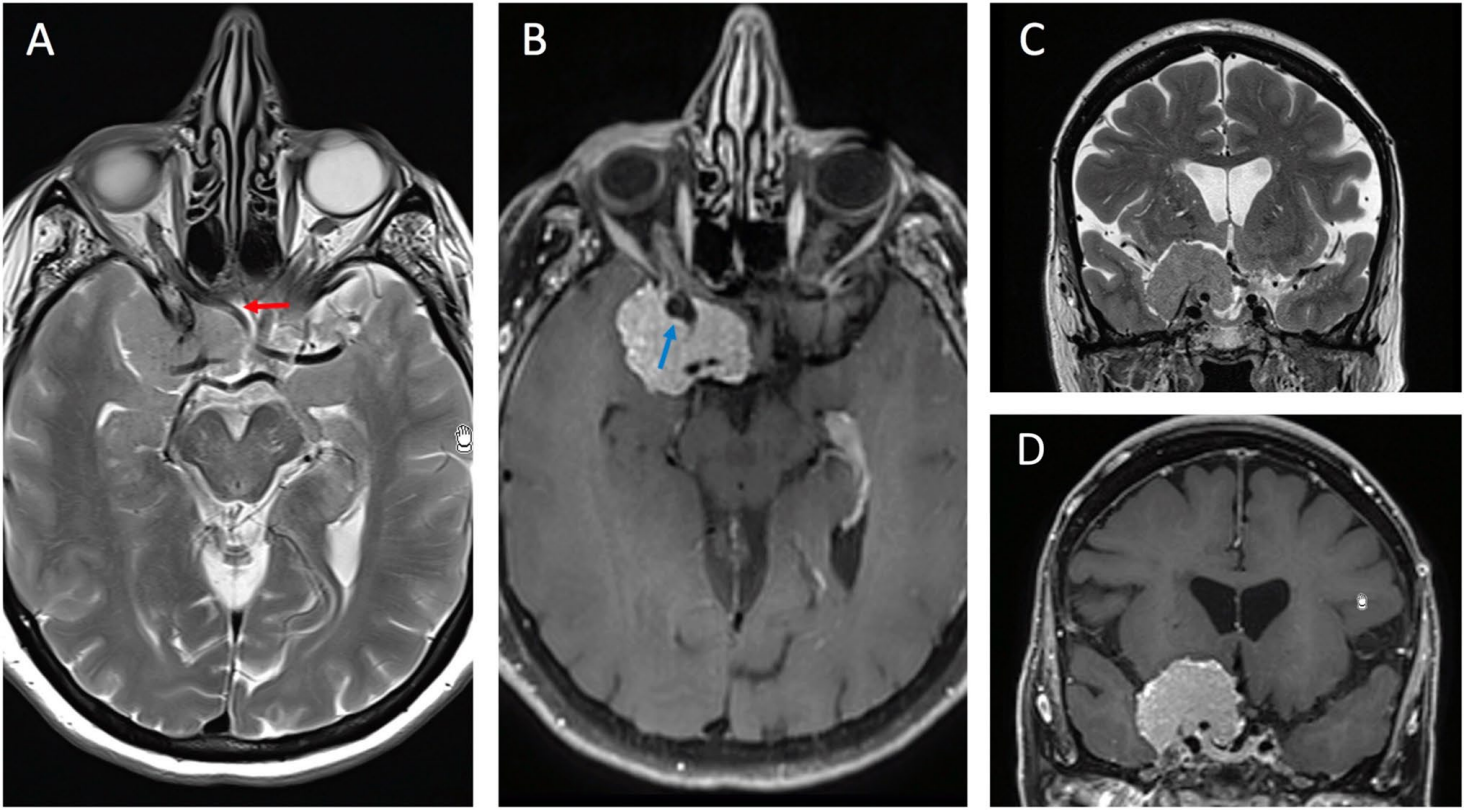
**A** and **B** Pre-operative T2W and Gd-enhanced T1W axial MR images showing a large Al-Mefty group I clinoidal meningioma with encasement of the carotid artery bifurcation (blue arrow) and compression of the ipsilateral optic nerve (red arrow). **C** and **D** T2W and Gd enhanced T1W coronal MR images showing the tumor extension within the cavernous sinus and the encasement of the carotid artery

The patient’s head is secured in a head holder and turned 20° to the opposite side in extension to allow frontal lobe retraction (Fig. 1). A frontotemporal craniotomy, flush to the anterolateral skull base, and an extradural clinoidectomy are performed [2, 8]. This allows early ON decompression, reduces effects of manipulation, early devascularization of the meningioma, and early identification of the ICA and ON.

A standard C-shaped basal durotomy is performed. The tumor is progressively detached from its implantation and debulked from within, paying careful attention to identifying and respecting the arachnoid plane. The important neurovascular structures like the ON and chiasm, the ICA and its branches and the oculomotor nerve, should be identified as early as possible and protected. Sectioning of falciform ligament and opening the dura of the proximal optic canal is a key step to remove the infiltrated dura and the tumor extending into the canal. This also allows a safer mobilization of the ON. The tumor capsule is progressively dissected from the surrounding structures respecting the arachnoid plane. The distal sylvian opening and dissection will depend on the encasement of the ICA bifurcation and proximal M1 segment.

In the absence of an arachnoid dissection plane between the tumor and the arterial wall, a thin tumor layer is left in place without an attempt at separating tumor off the vessel adventitia. No attempt is made for resection of the CS extensions in cases where oculomotor function is intact.

A particular attention is paid to the resection of the tumor component located inferolateral to the ON and inferomedial to the DDR. In this location, the unresected dura mater is carefully coagulated keeping under visual control the chiasma and its vascularizing perforators along with the ophthalmic artery. Attention is paid not to coagulate the dura covering the lateral wall of the CS to avoid injuring the nerves.

**Fig. 2:**
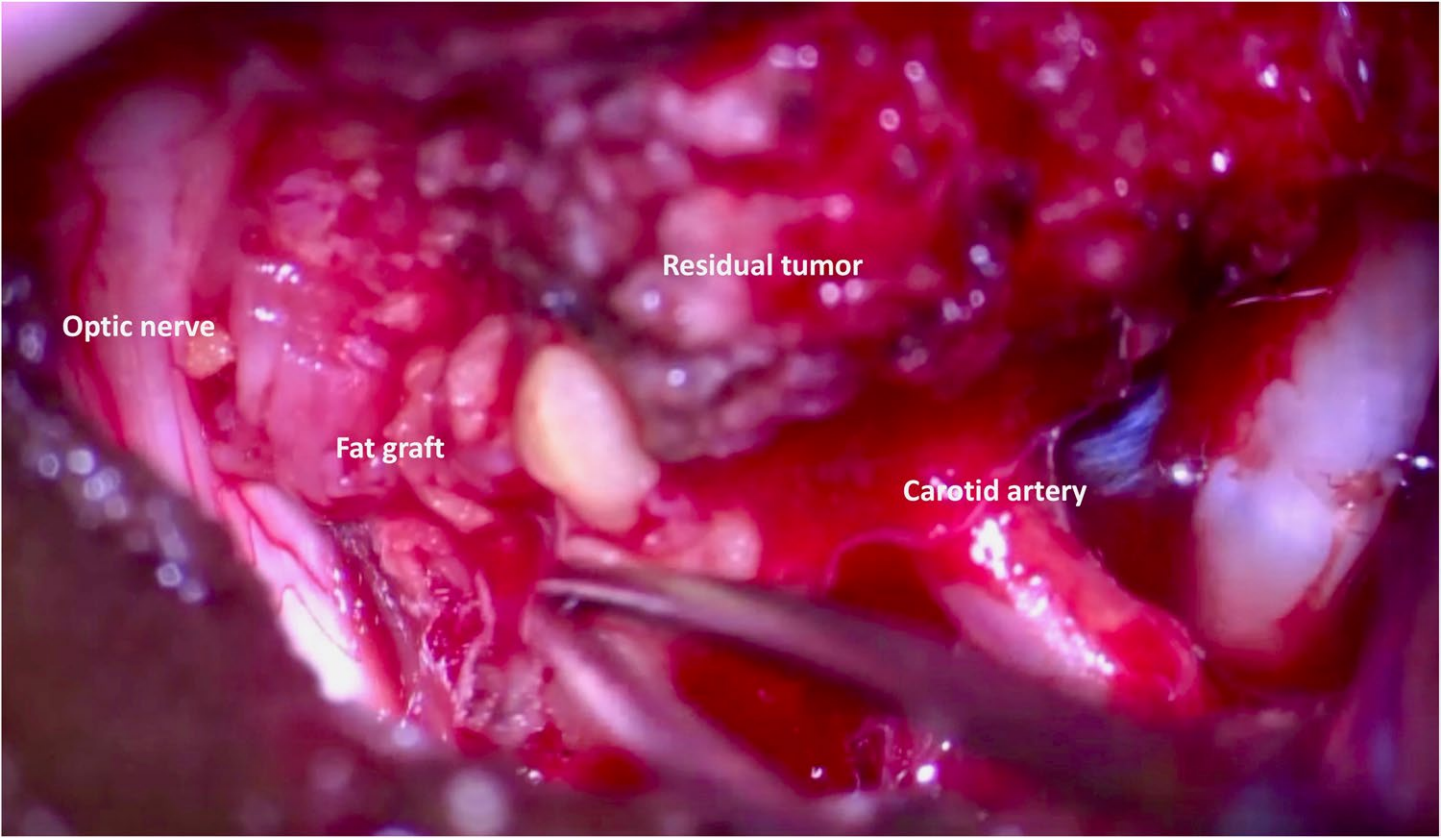
Intraoperative image showing the fat graft interposed between the optic nerve and the residual tumor within the cavernous sinus and distal dural ring

**Fig. 3:**
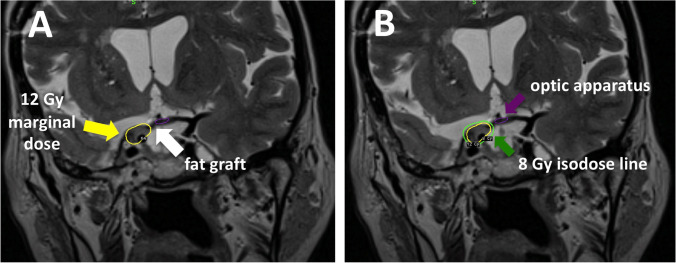
**A** and **B** Radiosurgery plan showing the yellow dosimetric curve of the tumor at 12 Gy. The green dosimetric curve shows the limit of the 8 Gray dose that passes through the fat graft and remains outside the contour of the optic pathway (violet dosimetric curve)

A fat graft, previously harvested from the infratemporal fossa, can be then placed between coagulated dural implantation sites and/or residual tumor and the optic apparatus (Fig. 2). This fat graft must be previously thinned and then positioned to protect the ON and anterior part of chiasm, which is then fixed in place with biological glue. This fat graft allows to maintain the distance gained at surgery to allow the delivery of radiosurgery usually after 3 months (Fig. 3).

A watertight dural closure is performed. The bone flap is replaced and secured using miniplates.

### Radiosurgery technique

We perform radiosurgery using Leksell Gamma Knife(GK ICON, Elekta Instruments, AB, Sweden) [9]. Marginal doses between 12 to 14 Gy (single fraction whenever feasible) are used, depending on the residual tumor volume and anatomical relationship between the residual tumor and optic apparatus. Thus, fat graft placed during surgery is crucial, allowing to deliver optimal therapeutic doses, while keeping a dose as low as possible to the optic pathways (8 to 12 Gy maximal dose) [5] (Fig. 3). The neurovascular contents of the CS are considered radioresistant, with no dose constraints. In current radiosurgery practice, risk of long-term modifications in the vessel wall of the ICA is considered exceptional [4].

## Indication

CS involvement and adventitial involvement of the ICA are the main factors limiting the extent of resection with gross total resection (GTR) achieved in only 11.8% [3, 7]. A recurrence rate of 60% after less-than-total resection has been reported, compared to a rate of about 5–10% in cases of GTR. In less than total resections, adjuvant radiosurgery represents an excellent method of treatment, to ensure local tumor control [7]. A delay of 3 months seems to be ideal based on our experience to maximize the benefit of the fat graft before its possible reabsorption. Moreover, the planning of the radiosurgery is easier when the analysis of the postoperative MR images is not hindered by signals related to blood and blood products in the early postoperative phase (Fig. 3).

## Limitations

Larger residual volumes will necessitate the use of hypofractionnated radiosurgery. This technique of near-total resection and adjuvant radiosurgery also has limited utility in en-plaque spheno-cavernous tumors due to the lack of clear dural margins in relation the ON trajectory following surgery. These patients are then treated with fractionated (or hypo-fractionated) radiation treatment plans to minimize complication risk and optimize tumor coverage [1].

## How to avoid complications

- An early OC decompression avoids nerve retraction and manipulation injury during tumor removal
- Coagulation of the unresected dura around the DDR should be carefully performed to avoid any lesion to the ophthalmic artery
- Extensive coagulation of the lateral wall of the CS should be avoided
- Avoiding an oversized fat graft is recommended to avoid iatrogenic compression
- Optimal marginal radiosurgical doses in single fraction between 12 to 15 Gy avoids neural complications
- The maximal dose received by the optic apparatus should be in excess of 8 Gy to avoid optic neuropathy, especially in patients with prior radiation [5]

## Specific postoperative considerations

**Fig. 4:**
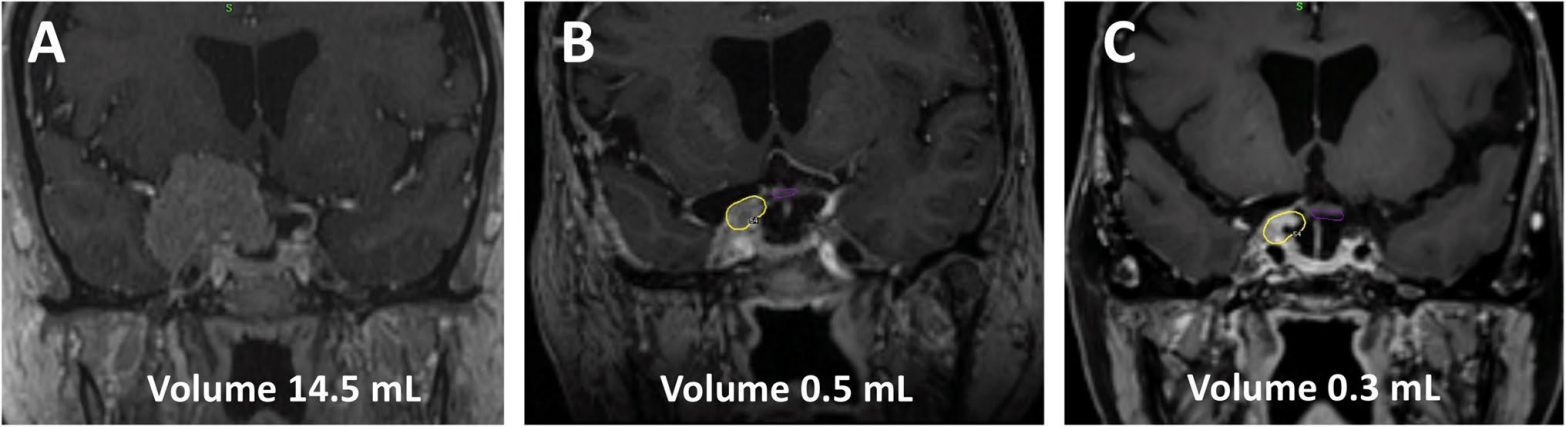
Gd-enhanced T1W coronal MR images showing the preoperative tumor (**A**) and residual volume at 3-month (**B**) and 1-year (C) follow-up

Early postoperative MRI is needed in the first 24/72 h to evaluate the presence of any residual tumor and also to define the location, size, and signals of the fat graft. The MRI at 3 months allows the determination of the feasibility of radiosurgery. Follow-up MRI after radiosurgery is performed at 6 months initially and then on a yearly basis (Fig. 4).

## Specific information to give to the patient about surgery and potential risks

Improvement of visual disturbance is expected in 48% of patients with a risk of deterioration of about 5% [3].

Local control after near-total resection and adjuvant radiosurgery has been reported to be favorable with 10-year PFS rates ranging from 69 to 97% [4].

## Ten key point summary

1. A pre-operative bone CT is fundamental to assess hyperostosis at the site of origin of the tumor and the anatomy of the ACP (its pneumatization, presence of a carotid-clinoid foramen and surrounding bony structures).
2. A skull base approach allows an early OC decompression and expands the surgical corridor, thereby avoiding brain and nerve retraction.
3. In the absence of an arachnoid dissection plane between the tumor and the arterial wall, a thin tumor layer is left in place.
4. No attempt is made for resection of the CS extensions in cases where oculomotor function is intact.
5. The unresected dura mater located inferolateral to the ON and inferomedial to the distal dural ring is carefully coagulated keeping under visual control the chiasma and its vascularizing perforators along with the ophthalmic artery.
6. The fat graft is placed between coagulated dural implantation sites and/or residual tumor and the optic apparatus.
7. Avoiding an oversized fat graft is recommended to avoid iatrogenic compression.
8. Optimal marginal radiosurgical doses in single fraction between 12 and 15 Gy avoids neural complications.
9. The maximal dose received by the optic apparatus should be in excess of 8 Gy to avoid optic neuropathy, especially in patients with prior radiation.
10. In case of an en-plaque spheno-cavernous tumor and/or lack of clear dural margins in relation to the ON trajectory, fractionated (or hypo-fractionated) radiation treatment plans are used to minimize complication risk and optimize tumor coverage.

## Supplementary information

Below is the link to the electronic supplementary material.

Supplementary file1 (MOV 48223 KB)
